# New Advances on Zika Virus Research

**DOI:** 10.3390/v11030258

**Published:** 2019-03-14

**Authors:** Luis Martinez-Sobrido, Fernando Almazán

**Affiliations:** 1 Department of Microbiology and Immunology, School of Medicine and Dentistry, University of Rochester, Rochester, New York, NY 14642, USA; 2 Department of Molecular and Cell Biology, Centro Nacional de Biotecnología (CNB-CSIC), Campus Universidad Autónoma de Madrid, 3 Darwin street, 28049 Madrid, Spain; falmazan@cnb.csic.es

Zika virus (ZIKV) is an emerging mosquito-borne member of the *Flaviviridae* family that has historically been known to cause sporadic outbreaks, associated with a mild febrile illness, in Africa and Southeast Asia. However, the recent outbreaks of ZIKV in the Americas and its association with severe neurological disorders, including fetal microcephaly, Guillain-Barré syndrome, and ocular abnormalities, have caused a great social and sanitary alarm. The significance of ZIKV in human health, together with a lack of approved therapeutic (antivirals) or prophylactic (vaccines) interventions, has triggered a global effort to develop effective countermeasures against this pathogen, which has the potential to affect millions of people worldwide.

Since the re-emergence of the virus in 2015 in Brazil, massive advances have been made in practically all areas of the biology of ZIKV. In this Special Issue, we have assembled a collection of 32 research papers and reviews that cover recent advances on ZIKV research in molecular biology, replication and transmission, virus-host interactions, pathogenesis, epidemiology, vaccine development, antivirals, and diagnosis.

The first part of this Special Issue focuses on the development of ZIKV reverse genetic approaches, which constitute a powerful tool to answer important questions on the biology of ZIKV and for vaccine development. This theme is covered by a complete review of all ZIKV reverse genetic systems developed in the last years (Ávila-Pérez et al. [1]) and two research papers describing the generation of a ZIKV infectious clone by the mutational silencing of cryptical bacterial promoters present in the viral genome (Münster et al. [2]) and a Tet-inducible ZIKV infectious clone (Zhang et al. [3]).

The second topic of the Special Issue addresses recent advances in viral replication and transmission and is covered by three research articles (Barnard et al. [4]; Mlera and Bloom [5]; and Oliveira et al. [6]).

The third topic, virus-host interactions, includes two comprehensive reviews, one describing the molecular insights into ZIKV-host interactions (Lee et al. [7]) and other discussing the type I interferon (IFN) antagonist mechanisms used by flaviviruses, with a focus on the non-structural (NS)5 protein (Thurmond et al. [8]). In addition, this topic includes five research manuscripts that describe the impact of viral and host genetic variations on ZIKV infection (Yun et al. [9]), the effect of ZIKV infection on Heme Oxygenase expression (Kalamouni et al. [10]), the different effects of ZIKV infection in placenta and microglia cells (Martinez-Viedma and Pickett [11]), the effect of permethrin resistance on the vector transcriptome after ZIKV infection (Zhao et al. [12]), and the microRNA and mRNA profiling in infected neurons (Azouz et al. [13]).

The fourth subject area address new advances in ZIKV pathogenesis. This theme is covered by two reviews that describe ZIKV pathogenesis in the male reproductive tract (Stassen et al. [14]) and the ocular abnormalities induced by flavivirus infection (Singh et al. [15]), and five research articles that define fetal brain infection with ZIKV isolates not associated with microcephaly (Setoh et al. [16]), the pathogenesis of Asian and African ZIKV isolates in Indian Rhesus macaques (Rayner et al. [17]), the consequences of ZIKV infection in human pluripotent stem cell-derived neural progenitor cells and neurons (Goodfellow et al. [18]), the effect of a single mutation in the NS2A protein in virus pathogenesis (Márquez-Jurado et al. [19]), and the roles of the premembrane (prM) and envelop (E) proteins in ZIKV-mediated infection and neurocytotoxicity (Li et al. [20]).

The fifth section in the Special Issue covers the new advances in epidemiology and virus evolution, including a manuscript describing the evolutionary insight of ZIKV strains isolated in Latin America (Simón et al. [21]).

The next section focuses on ZIKV vaccines and antivirals, and contains three review documents (Garg et al. [22]; Alves et al. [23]; Saiz et al. [24]) and two research articles that describe the antiviral effect of silvestrol (Elgner et al. [25]) and oxysterol 7-ketocholesterol (Willard et al. [26]) in ZIKV replication.

The last section in this Special Issue covers new advances in the molecular diagnostic of ZIKV, and includes a comprehensive review (Mantke et al. [27]) and five research articles that describe the development and characterization of several ZIKV diagnostic methods (Bhadra et al. [28]; de Ory et al. [29]; Zhang et al. [30]; Taylor et al. [31]; Amaro et al. [32]).

We would like to thank all contributing authors for their participation, effort and hard work in putting together this Special Issue. We would also like to thank the Editorial Office at *Viruses* for all the help, support, and advice with this Special Issue. We hope this Special Issue offers a comprehensive view of the recent advances in ZIKV research and stimulates research for future studies aimed at understanding ZIKV evolution, virus-host-interaction, pathogenesis, and the development of effective countermeasures to combat ZIKV infection.
